# Supplementary material for: Analysis of the Mechanism of Wood Vinegar and Butyrolactone Promoting Rapeseed Growth and Improving Low-Temperature Stress Resistance Based on Transcriptome and Metabolomics
Source: Int J Mol Sci. 2024 Sep 9;25(17):9757. doi: 10.3390/ijms25179757 (PMC11395900; doi:10.3390/ijms25179757)
Supplement: Supplementary file 1 [file ijms-25-09757-s001.zip › Table S1-S4 and Figure S1-S4.pdf]

Table S1. Main components of wood vinegar.

Retention Time (min)	Compounds	Relative Content (%)	Formula	Molar Mass (g mol^{-1})
	**Organic acids**	**26.97**		
1.93	Formic acid	0.39	CH_2O_2	46
3.16	Acetic acid	22.99	$C_2H_4O_2$	60
4.76	Propanoic acid	0.38	$C_3H_6O_2$	74
5.59	2-Hydroxy-2-met-propanoic acid	0.11	$C_5H_{10}O_3$	118
9.93	Butyric acid	2.55	$C_4H_8O_2$	88
7.21	2-Oxo-n-valericacid	0.55	$C_5H_8O_3$	116
	**Ketones**	**10.53**		
2.24	2-Butanone	0.37	C_4H_8O	72
3.58	Acetoin	0.3	$C_4H_8O_2$	88
4.80	1-Hydroxy-2-butanone	0.28	$C_4H_8O_2$	88
5.31	Cyclopentanone	0.62	C_5H_8O	84
6.15	1-Hydroxy-3-methyl-2-butanone	0.21	$C_5H_{10}O_2$	102
6.50	2-Cyclopenten	1.61	C_5H_6O	82
8.81	2-Methyl-2-cyclopenten-1-one	1.44	C_6H_8O	96
12.47	2,5-Dihydro-3,5-dimeth-2-furanone	0.66	$C_6H_8O_2$	112
13.80	2,3-Dimeth-2-cyclopenten-1-one	1.25	$C_7H_{10}O$	110
13.96	3-Methyl-1,2-cyclopentanedione	1.14	$C_6H_8O_2$	112
15.85	3-Ethyl-2-methyl-2-cyclopenten-1-one	0.22	$C_7H_{10}O_2$	126
20.48	2-Hydroxy-3-propyl-2-cyclopenten-1-one	0.38	$C_8H_{12}O_2$	140
29.38	1-(4-Hydroxy-3-methoxyphenyl)-ethanone	0.48	$C_9H_{10}O_3$	166
30.66	1-(4-Hydroxy-3-methoxyphenyl)-2-propanone	1.29	$C_{10}H_{12}O_3$	180
36.34	1-(4-Hydroxy-3,5-dimethoxyphenyl)-ethanone	0.28	$C_{10}H_{12}O_4$	196
	**Esters**	**3.85**		
2.37	Ethylacetate	0.67	$C_4H_8O_2$	88
9.24	Gamma-butyrolactone	2.34	$C_4H_6O_2$	86
11.32	Methyl2-furoate	0.09	$C_6H_6O_3$	126
14.73	2,6-Dimethyl-1-cyclohexen-1-ylacetate	0.75	$C_{10}H_{16}O_2$	168
	**Furan derivatives**	**1.48**		
5.51	Tetrahydro-2-furanol	0.41	$C_4H_8O_2$	88
4.33	2-Methoxytetrahydrofuran	0.19	$C_5H_{10}O_2$	102
8.98	1-(2-Furanyl)-ethanone	0.88	$C_6H_6O_2$	110
	**Alkanes compounds**	**3.36**		
5.79	Methoxymethyl-oxirane	0.07	$C_4H_8O_2$	88
9.17	3-Bromo-pentane	0.59	$C_5H_{11}Br$	150
15.63	Bicyclo [2.2.2] octane	2.7	C_8H_{14}	110
	**Aldehydes**	**1.72**		
10.85	5-Methyl-2-furancarboxaldehyde	0.19	$C_6H_6O_2$	110
26.77	Vanillin	1.53	$C_8H_8O_3$	152
	**Phenols and derivatives**	**43.19**		
11.95	Phenol	5.91	C_6H_6O	94
14.64	O-cresol	2.41	C_7H_8O	108
15.48	3-Methylphenol	3.74	C_7H_8O	108
15.73	Guaiacol	2.75	$C_7H_8O_2$	124
18.03	2,4-Dimethylphenol	0.89	$C_8H_{10}O$	122
18.11	2,5-Dimethylphenol	0.39	$C_8H_{10}O$	122
18.65	4-Ethyl-phenol	0.47	$C_8H_{10}O$	122
18.82	3,5-Dimethylphenol	0.37	$C_8H_{10}O$	122
19.03	2,3-Dimethylphenol	0.22	$C_8H_{10}O$	122
19.50	2-Methoxy-4-methylphenol	2.04	$C_8H_{10}O_2$	138
19.60	3,4-Dimethylphenol	0.27	$C_8H_{10}O$	122
20.01	Catechol	8.95	$C_6H_6O_2$	110
22.07	4-Methyl-1,2-benzenediol	4.81	$C_7H_8O_2$	124
22.51	4-Ethyl-2-methoxyphenol	1.32	$C_9H_{12}O_2$	152
25.14	2,6-Dimethoxy-phenol	3.56	$C_8H_{10}O_3$	154
25.23	2,5-Dimethyl-1,4-benzenediol	0.9	$C_8H_{10}O_2$	138
25.37	3,4-Dimethoxy-phenol	0.29	$C_8H_{10}O_3$	154
26.29	4-Ethylcatechol	1.8	$C_8H_{10}O_2$	138
28.13	1,2,4-Trimethoxybenzene	1.65	$C_9H_{12}O_3$	168
30.44	1,2,3-Trimethoxy-5-methyl-benzene	0.45	$C_{10}H_{14}O_3$	182
	**Nitrogen compounds**	**1.46**		
21.93	Pyridine	1.46	C_5H_5N	79

Table S2. Primer sequences for fluorescence quantitative PCR.

Gene	Forward Primer Sequence	Reverse Primer Sequence
BnaA01G0007100ZS	CCTTCAGACCCACCATCTGTTCT	GGGTCTCCAACAGTGGCGT
BnaA01G0008900ZS	TTGGAAGCTCATCATGTGTTGCTC	ACGAGAATGTGTATTACGGGACTGT
BnaA01G0329500ZS	TGCACCTCCGTGGCTTCT	TGCTTGGGCCACGACCAT
BnaA02G0103500ZS	GAGGAAAGGGAGGAAAGGGTTTGG	TAGCGTGCTCAGTGTATGTCACAG
BnaA02G0317700ZS	ATTCATGGCGCTTTTCATAATATCCCAA	GATTAAAACATCCAGGAGGAACAGGAGT
BnaA04G0140100ZS	GCATGGGCCACCGACGA	GCCGCTTCCACGGGACT
BnaA10G0113800ZS	TGGTTGTAACCGCCGCGA	GCCACAATTGGTCCCTAGAGC
BnaC03G0034400ZS	TGGATACACCCAAGGGTTCCT	TCCCTGATCTGTATTGGGTTCCA
BnaA01G0023100ZS	ACCCTCAAGGTTGCTCCCTC	TGGTGGAGGCTTGGACCATC
BnaA01G0122600ZS	ACCATGAGAACGTCATTGACCAC	TGGCAACAGATGAAGAGGCGA
BnaA01G0298400ZS	TGCAGTCAGTCTTCGCCTG	TGGAGGAGGTGGTGGCG
BnaA10G0199800ZS	GCCGCGCAGGGCTTATC	TGGCTTGTTACTCAAATCCACCA
BnaC01G0367700ZS	CACCTCCTCCACCTCCTACTAAAC	CAAGATCGCAGTCGTACCAAGGAA
BnaA01G0004300ZS	AGTGGAGCAAGGGGAAGCA	AGTGAGCAGCGACCATCCT
BnaA01G0009500ZS	GGCAAGGACAGAAACTAGCGG	TGCTTCTCCGCTTCACTTGGT
BnaA04G0030300ZS	TCTCAGCCTTCTAATGGCTTCCA	TCGCCATTGGGAACGTCG
BnaA04G0253700ZS	TGCTAACAAACCCATGATCCTTCA	AGGCTCTCCAGAACCATCAATCAA
BnaC03G0546100ZS	TGGTCTCTTTCCCTTTGGCGT	TCCGACCTTGTGCTGATGCT
BnaA01G0003600ZS	GCTTTCCTTCTCCCTTCTCCTCC	TCGCCTCAGATTGGCTTTGAA
BnaA01G0011500ZS	ACGGTGAAGGCAACGACGA	AGTGGAACCGTCGGCCT
BnaA01G0019000ZS	AGCTCCTTATCGAGTTCGTGCT	AGAAACAAGGCCAAAGCTGCAA
BnaA03G0002200ZS	TGGCCTCCAAGGCGCTC	TGGCCAGATCGGCTTTCTTCA
BnaA03G0517000ZS	AGCTTGTTCCTGGCAACTCAG	GGAAGTTCGCTGTTGGGTCG
BnaA04G0006100ZS	ACGAAAATGAGCTAGTGGAGTCAGT	ATACTCGCTCACACTGCCTAGCAA
BnaA08G0288400ZS	GGCGTTGGCTGGAATCGC	TGCTTAAGCGCACTCTCCGT
BnaA09G0006300ZS	TACCACGACAAGGAGTTCAGATCC	GTTGCTTTGAGGAACCATTTGCCT
BnaC03G0004200ZS	TCTGAGCTTCTCAAGGCACACT	TTGCTCGTTAATGGCAGCCT
Actin7_141	GGAAGCTCCTGGAATCCATGAGA	TCTTTGCTCATACGGTCAGCAATTCC

Table S3. Sequencing Data Statistics.

Sample	Clean Reads Pairs	Clean Base (bp)	Q20 (%)	Q30 (%)	GC (%)
CK12h-1	25,515,031	7,388,056,111	98.10;95.16	93.92;87.39	48.50;48.47
CK12h-2	25,281,863	7,429,036,971	97.97;97.13	93.67;91.57	48.44;48.47
CK12h-3	21,299,057	6,214,976,134	97.95;97.19	93.63;91.78	48.53;48.47
CK24h-1	24,291,452	7,060,458,224	98.04;97.50	93.82;92.45	48.84;48.82
CK24h-2	20,614,680	5,933,766,708	98.09;97.46	93.98;92.39	48.74;48.81
CK24h-3	23,061,799	6,710,906,806	98.10;97.32	93.99;92.06	49.01;49.00
CK6h-1	21,783,825	6,423,459,853	97.94;97.33	93.60;92.08	48.72;48.71
CK6h-2	24,743,021	7,246,528,162	98.00;96.97	93.74;91.24	48.68;48.61
CK6h-3	24,769,942	7,240,424,855	97.99;97.49	93.71;92.40	48.77;48.66
CK72h-1	22,441,339	6,573,237,335	98.10;97.54	93.98;92.55	48.88;48.89
CK72h-2	22,501,514	6,601,117,487	98.09;97.51	93.97;92.45	48.91;48.80
CK72h-3	23,980,181	7,024,608,706	98.07;97.53	93.90;92.49	48.70;48.65
D12h-1	24,741,354	7,177,305,378	97.96;97.47	93.63;92.37	48.38;48.35
D12h-2	25,242,426	7,370,930,899	97.97;97.46	93.67;92.33	48.45;48.39
D12h-3	23,274,042	6,780,570,634	98.00;97.41	93.71;92.22	48.31;48.27
D24h-1	23,457,823	6,855,153,268	98.00;96.92	93.74;91.15	48.73;48.63
D24h-2	23,576,227	6,866,673,015	98.06;97.17	93.89;91.68	48.68;48.64
D24h-3	24,038,056	6,992,125,545	98.05;97.65	93.87;92.80	49.01;48.89
D6h-1	27,369,800	7,944,630,568	99.22;98.24	97.35;94.97	48.53;48.44
D6h-2	21,128,104	6,138,078,826	99.20;98.20	97.27;94.86	48.70;48.49
D6h-3	21,666,419	6,326,980,872	97.92;96.63	93.52;90.55	48.08;48.06
D72h-1	24,651,246	7,142,484,020	98.10;97.31	93.98;91.99	48.63;48.68
D72h-2	22,599,790	6,565,217,652	98.09;97.23	93.97;91.85	49.01;48.96
D72h-3	23,631,945	6,970,922,594	98.08;96.94	93.92;91.19	48.71;48.83
M12h-2	21,278,167	6,203,467,801	97.95;96.97	93.64;91.27	48.43;48.43
M12h-3	25,299,029	7,348,590,355	97.97;97.35	93.66;92.10	48.51;48.45
M24h-1	20,319,026	5,938,512,580	98.02;97.48	93.78;92.41	48.57;48.60
M24h-2	23,747,852	6,945,398,437	98.07;97.32	93.91;92.01	48.67;48.57
M24h-3	24,508,264	7,158,932,516	98.07;97.43	93.90;92.27	48.57;48.34
M6h-1	21,006,066	6,162,190,431	97.95;97.10	93.62;91.57	48.63;48.56
M6h-2	24,560,922	7,184,490,361	97.99;97.13	93.70;91.58	48.59;48.56
M6h-3	24,940,258	7,261,083,934	97.99;97.03	93.69;91.34	48.52;48.47
M72h-1	23,715,688	6,916,415,892	98.10;97.43	93.97;92.26	48.84;48.81
M72h-2	23,766,292	6,891,002,976	98.10;97.34	93.97;92.07	48.76;48.74
M72h-3	24,367,056	7,133,338,846	98.08;97.46	93.92;92.35	48.72;48.80

Note: CK, Control. M, Wood vinegar. D, Butyrolactone.

Table S4. Comparison of sequencing data and reference genome results statistics.

Sample	Total Reads	Total mapped reads	Uniq mapped reads	Multiple mapped reads
CK12h-1	25,515,031	22,448,256(87.98%)	20,662,620(80.98%)	1,785,636(7.00%)
CK12h-2	25,281,863	22,666,773(89.66%)	20,898,019(82.66%)	1,768,754(7.00%)
CK12h-3	21,299,057	19,121,903(89.78%)	17,641,400(82.83%)	1,480,503(6.95%)
CK24h-1	24,291,452	21,695,569(89.31%)	19,797,955(81.50%)	1,897,614(7.81%)
CK24h-2	20,614,680	18,630,355(90.37%)	17,027,011(82.60%)	1,603,344(7.78%)
CK24h-3	23,061,799	20,885,123(90.56%)	19,182,632(83.18%)	1,702,491(7.38%)
CK6h-1	21,783,825	19,620,079(90.07%)	18,114,554(83.16%)	1,505,525(6.91%)
CK6h-2	24,743,021	22,159,761(89.56%)	20,455,984(82.67%)	1,703,777(6.89%)
CK6h-3	24,769,942	22,413,551(90.49%)	20,642,628(83.34%)	1,770,923(7.15%)
CK72h-1	22,441,339	20,401,177(90.91%)	18,745,928(83.53%)	1,655,249(7.38%)
CK72h-2	22,501,514	20,444,383(90.86%)	18,799,069(83.55%)	1,645,314(7.31%)
CK72h-3	23,980,181	21,616,054(90.14%)	19,837,962(82.73%)	1,778,092(7.41%)
D12h-1	24,741,354	22,052,448(89.13%)	20,100,923(81.24%)	1,951,525(7.89%)
D12h-2	25,242,426	22,553,084(89.35%)	20,560,694(81.45%)	1,992,390(7.89%)
D12h-3	23,274,042	20,869,165(89.67%)	19,181,392(82.42%)	1,687,773(7.25%)
D24h-1	23,457,823	21,187,699(90.32%)	19,502,660(83.14%)	1,685,039(7.18%)
D24h-2	23,576,227	21,208,809(89.96%)	19,497,760(82.70%)	1,711,049(7.26%)
D24h-3	24,038,056	21,877,447(91.01%)	19,933,809(82.93%)	1,943,638(8.09%)
D6h-1	27,369,800	24,501,689(89.52%)	22,564,656(82.44%)	1,937,033(7.08%)
D6h-2	21,128,104	18,873,887(89.33%)	17,373,635(82.23%)	1,500,252(7.10%)
D6h-3	21,666,419	19,292,480(89.04%)	17,912,487(82.67%)	1,379,993(6.37%)
D72h-1	24,651,246	22,341,956(90.63%)	20,434,479(82.89%)	1,907,477(7.74%)
D72h-2	22,599,790	20,172,142(89.26%)	18,170,237(80.40%)	2,001,905(8.86%)
D72h-3	23,631,945	21,268,042(90.00%)	19,755,058(83.59%)	1,512,984(6.40%)
M12h-1	23,207,516	20,777,234(89.53%)	19,168,627(82.60%)	1,608,607(6.93%)
M12h-2	21,278,167	19,036,394(89.46%)	17,528,730(82.38%)	1,507,664(7.09%)
M12h-3	25,299,029	22,867,853(90.39%)	20,971,814(82.90%)	1,896,039(7.49%)
M24h-1	20,319,026	18,270,971(89.92%)	16,828,449(82.82%)	1,442,522(7.10%)
M24h-2	23,747,852	21,549,730(90.74%)	19,937,525(83.96%)	1,612,205(6.79%)
M24h-3	24,508,264	21,610,949(88.18%)	19,964,802(81.46%)	1,646,147(6.72%)
M6h-1	21,006,066	18,753,069(89.27%)	17,320,736(82.46%)	1,432,333(6.82%)
M6h-2	24,560,922	22,081,359(89.90%)	20,378,261(82.97%)	1,703,098(6.93%)
M6h-3	24,940,258	22,301,629(89.42%)	20,520,333(82.28%)	1,781,296(7.14%)
M72h-1	23,715,688	21,309,933(89.86%)	19,455,577(82.04%)	1,854,356(7.82%)
M72h-2	23,766,292	21,614,270(90.95%)	19,750,534(83.10%)	1,863,736(7.84%)
M72h-3	24,367,056	22,087,235(90.64%)	20,286,284(83.25%)	1,800,951(7.39%)

Note: CK, Control. M, Wood vinegar. D, Butyrolactone.

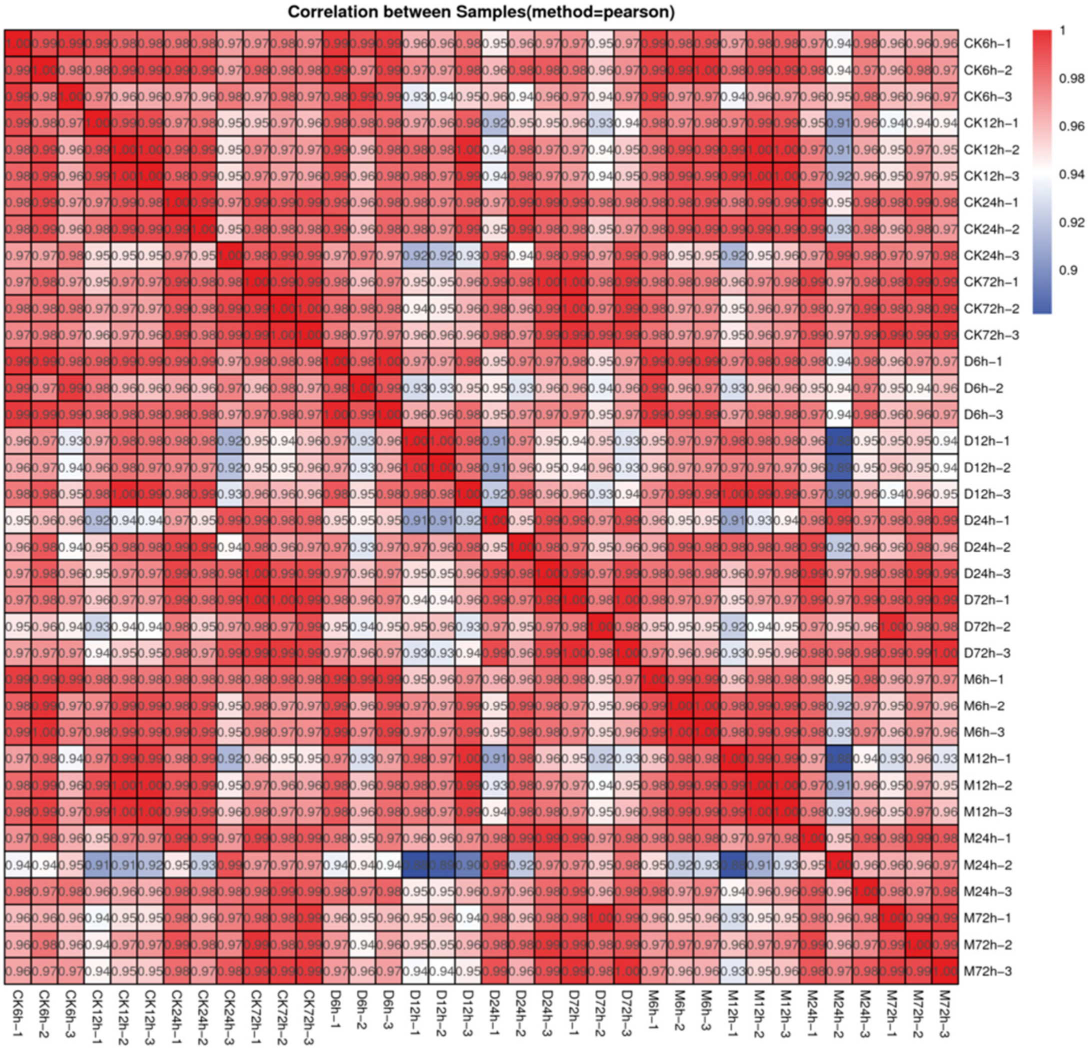

Figure S1. Heat map of correlation between samples. CK, Control. M, Wood vinegar. D, Butyrolactone.

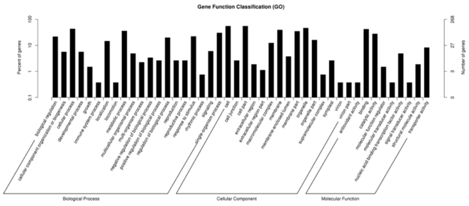

M _ up. GO _ classification _ bar

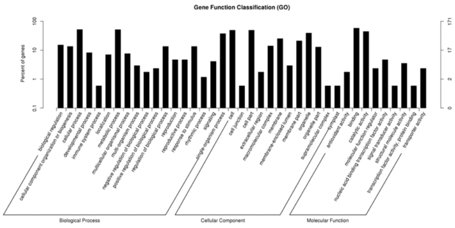

M _ down. GO_ classification_ bar

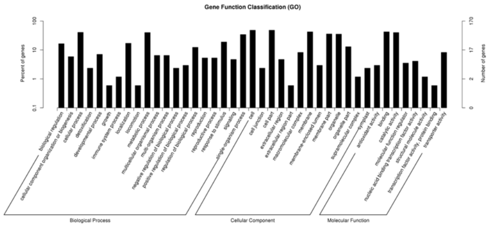

D _ up. GO _ classification_ bar

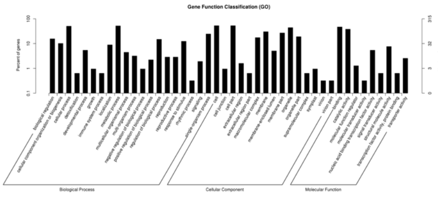

D _ down. GO_ classification_ bar

Figure S2. GO classification of differentially expressed genes. CK, Control. M, Wood vinegar. D, Butyrolactone.

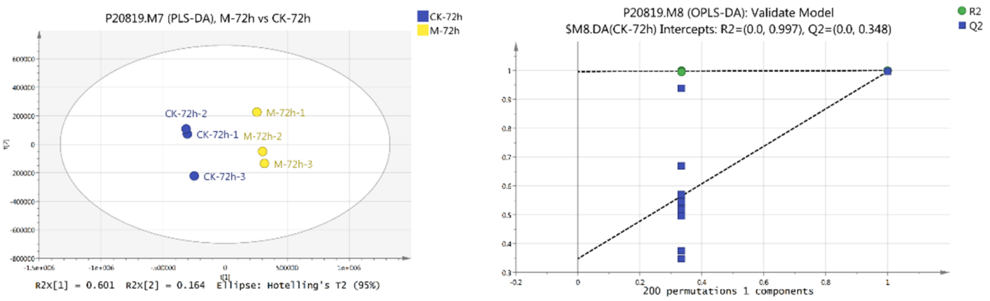

Figure S3. Partial least squares discriminant analysis and orthogonal partial least squares discriminant analysis of metabolites treated with wood vinegar solution. CK, Control. M, Wood vinegar. D, Butyrolactone.

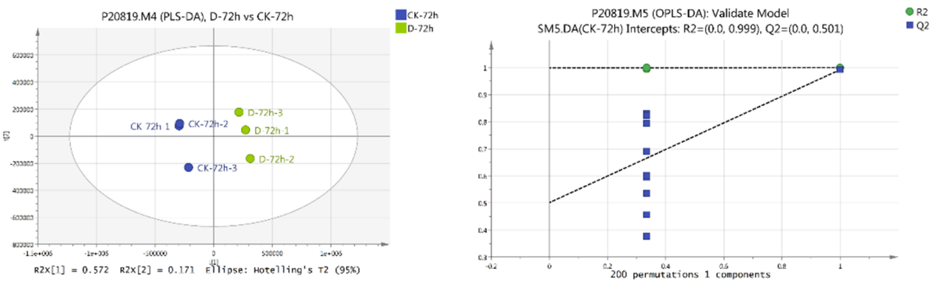

Figure S4. Partial least squares discriminant analysis and orthogonal partial least squares discriminant analysis of metabolites treated with butyrolactone. CK, Control. M, Wood vinegar. D, Butyrolactone.
